# The FGF/FGFR System in Breast Cancer: Oncogenic Features and Therapeutic Perspectives

**DOI:** 10.3390/cancers12103029

**Published:** 2020-10-18

**Authors:** Maria Francesca Santolla, Marcello Maggiolini

**Affiliations:** Department of Pharmacy, Health and Nutritional Sciences, University of Calabria, 87036 Rende, Italy; mariafrancesca.santolla@unical.it

**Keywords:** breast cancer, FGF/FGFR system, tumor microenvironment, oncogenic signaling, targeted therapies

## Abstract

**Simple Summary:**

The fibroblast growth factor/fibroblast growth factor receptor (FGF/FGFR) system represents an emerging therapeutic target in breast cancer. Here, we discussed previous studies dealing with *FGFR* molecular aberrations, the alterations in the FGF/FGFR signaling across the different subtypes of breast cancer, the functional interplay between the FGF/FGFR axis and important components of the breast microenvironment, the therapeutic usefulness of FGF/FGFR inhibitors for the treatment of breast cancer.

**Abstract:**

One of the major challenges in the treatment of breast cancer is the heterogeneous nature of the disease. With multiple subtypes of breast cancer identified, there is an unmet clinical need for the development of therapies particularly for the less tractable subtypes. Several transduction mechanisms are involved in the progression of breast cancer, therefore making the assessment of the molecular landscape that characterizes each patient intricate. Over the last decade, numerous studies have focused on the development of tyrosine kinase inhibitors (TKIs) to target the main pathways dysregulated in breast cancer, however their effectiveness is often limited either by resistance to treatments or the appearance of adverse effects. In this context, the fibroblast growth factor/fibroblast growth factor receptor (FGF/FGFR) system represents an emerging transduction pathway and therapeutic target to be fully investigated among the diverse anti-cancer settings in breast cancer. Here, we have recapitulated previous studies dealing with *FGFR* molecular aberrations, such as the gene amplification, point mutations, and chromosomal translocations that occur in breast cancer. Furthermore, alterations in the FGF/FGFR signaling across the different subtypes of breast cancer have been described. Next, we discussed the functional interplay between the FGF/FGFR axis and important components of the breast tumor microenvironment. Lastly, we pointed out the therapeutic usefulness of FGF/FGFR inhibitors, as revealed by preclinical and clinical models of breast cancer.

## 1. Introduction 

Breast cancer is the most frequently diagnosed malignancy and the second leading cause of cancer death in women worldwide [1]. The high incidence of this disease, the complex mechanisms involved in its progression, as well as the resistance to chemotherapy underline the need to better elucidate the molecular features fueling breast tumors. Over the past few years, substantial advances have been made in the discovery of new drugs for the treatment of this malignancy. The improved understanding of breast cancer heterogeneity has allowed the development of more effective and individualized therapeutic approaches [2]. Nevertheless, only a limited number of targeted therapies have been approved and are currently used in breast cancer patients. In this context, anti-human epidermal growth factor receptor 2 (HER2) therapeutics such as trastuzumab, lapatinib, and pertuzumab have been proposed and used even in combination therapies [3,4,5]; nevertheless, the lack of responsiveness has been also observed [6]. Indeed, the intrinsic and acquired resistance to classical therapeutic agents and/or novel targeted drugs still represents a main problem in the treatment of breast cancer and a major cause of relapse and cancer death. Therefore, recent advances in the discovery of druggable kinase alterations are fostering the development of novel therapeutic strategies towards a better clinical management of breast cancer patients.

In this context, the fibroblast growth factor/fibroblast growth factor receptor (FGF/FGFR) system has recently attracted increasing interest due to its role in tumor growth, metastasis, and resistance to anti-cancer therapies [7,8]. For instance, aberrant FGF/FGFR activation may occur in both a ligand-dependent and ligand-independent manner in several tumors, including breast cancer [8,9,10,11,12,13]. Experimental and clinical evidence has also shown that deregulated FGF/FGFR signaling may elicit an oncogenic action [14,15,16,17], though some reports have indicated that the FGF/FGFR axis may exert an anticancer action in certain contexts [18,19]. Of note, various studies have emphasized the role of the transduction network triggered by the FGF/FGFR system towards a stimulatory interaction between tumor and stromal cells [20,21]. The aforementioned observations, together with the involvement of FGF/FGFR signaling in resistance to endocrine therapies [22,23], have suggested that the FGF/FGFR system may be considered a promising therapeutic target in order to establish more comprehensive anti-cancer strategies.

Here, we recapitulate the recent discoveries regarding FGF/FGFR action in breast cancer. In particular, we focus on the *FGFR* genetic aberrations that may occur in breast cancer subtypes and the multifaceted interactions prompted by the FGF/FGFR axis in breast tumor stroma. In addition, we highlight the promising therapies targeting the FGF/FGFR system towards a better outcome for breast cancer patients. 

## 2. The FGF/FGFR System 

### 2.1. Receptors and Ligands

The human FGFR family includes four highly conserved receptor tyrosine kinases (RTKs)—namely, FGFR1, FGFR2, FGFR3, and FGFR4—that are encoded by distinct genes, and an additional receptor named FGFR5 (also known as FGFRL1) which lacks the intracellular kinase domain [11]. On the basis of this peculiar feature, FGFR5 acts as a decoy receptor that, when binding to FGF ligands, may prevent their interaction with other FGFRs [24]. FGFR1-4 contain the classical extracellular domain, a transmembrane domainm and a tyrosine kinase cytoplasmic domain. The extracellular region encompasses three immunoglobulin-like subdomains (I, II, and III) and an acid box, which is typically located between the subdomains I and II, whereas the FGF ligand-binding site relies on the II and III subdomains [25]. The transmembrane region is made up of a single α-helix and the intracellular tyrosine kinase domain exhibits the canonical bilobed architecture of the protein kinases [26]. 

The FGF ligands belong to a family of 22 conserved molecules. The FGFs 1–10 and the FGFs 16–23 are secreted signaling molecules that interact with the FGFRs, while FGFs 11–14 do not act as FGFR ligands and their functions remain to be fully understood [11]. The FGF ligands can be classified into subfamilies according to the mechanism of action. Many FGFs act in an autocrine and/or paracrine manner, binding to FGFRs together with heparan sulfate proteoglycans (HSPGs), whereas FGF 19, FGF 21, and FGF 23 behave like endocrine ligands [27,28]. The endocrine FGFs lack the HSPG binding; consequently, they may diffuse from the site of production to the blood stream. Recruiting as cofactors certain members of the Klotho family, the endocrine FGFs contribute to maintaining whole-body homeostasis through FGFR-mediated signaling [29,30,31]. 

### 2.2. FGF/FGFR Signaling Cascade

**Figure 1:**
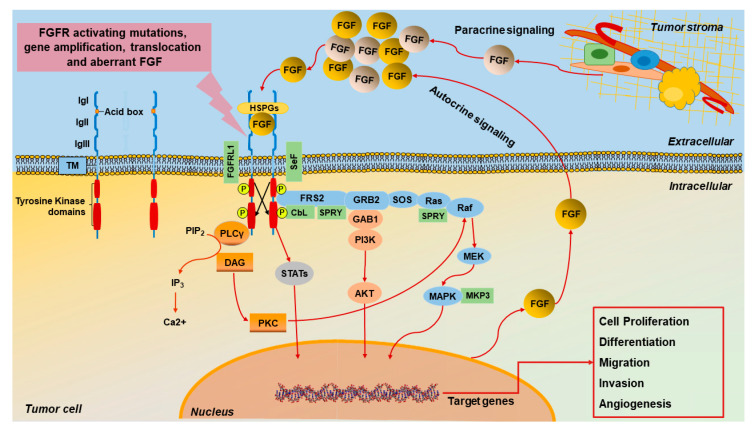
Schematic representation of aberrant FGF/FGFR signaling in breast cancer. The FGFR comprises three extracellular immunoglobulin-like subdomains (IgI, IgII, and IgIII), a transmembrane α-helix, and an intracellular bilobed cytoplasmic tyrosine kinase domain. IgI and IgII are separated by an acidic box. FGFs, which are secreted by tumor cells and/or the stromal compartment, bind to membrane-anchored FGFR monomers, then induce FGFR dimerization and the phosphorylation of the tyrosine kinase domains. The binding of FGFs to FGFRs is stabilized by heparan sulphate proteoglycans (HSPGs). Subsequently, the complex leads to the docking of adapter proteins and the activation of downstream pathways. For instance, ligand-stimulated FGFRs phosphorylate the FGFR-associated cytosolic docking protein FRS2. Once phosphorylated, FRS2 recruits Son of Sevenless adapter protein (SOS) and growth factor receptor-bound protein (GRB2) to activate rat sarcoma (RAS) and the downstream Rapidly Accelerated Fibrosarcoma (RAF) and MAPK/ERK Kinase (MEK) pathway. A different complex involves GRB2-associated binding protein 1 (GAB1), which recruits phosphoinositide 3-kinase (PI3K) that activates the Protein Kinase B (AKT) pathway. Phospholipase Cγ (PLCγ) hydrolyzes phosphatidylinositol-4,5-bisphosphate (PIP2) to phosphatidylinositol (3,4,5)-triphosphate (PIP3) and diacylglycerol (DAG). PIP3 releases calcium, whereas DAG activates protein kinase C, which helps to strengthen the activation of the MAPK pathway by phosphorylating RAF in a RAS-independent manner. Other cascades may also be activated by FGFRs, such as the signal transducer and activator of transcription (STAT)-dependent signaling. The aforementioned effectors regulate, in turn, gene expression as well as cell proliferation, migration, invasion, and angiogenesis. FGF/FGFR signaling may be down-regulated by receptor internalization or through negative regulators such as FGFR-like 1 (FGFRL1), with a similar expression to FGF (SEF), Sprouty (SPRY), Casitas B-lineage Lymphoma (CbL), and MAPK phosphatase 3 (MKP3).

Upon ligand activation, the FGFRs dimerize and the intracellular tyrosine kinase domains become phosphorylated and engage with various downstream proteins such as FGFR substrate 2 (FRS2), phospholipase Cγ (PLCγ), as well as diverse transduction pathways such as RAS-mitogen-activated protein kinase (MAPK), phosphatidylinositol 3-kinase (PI3K)–AKT, inositol-1,4,5-trisphosphate (IP3)–Ca^2+^, diacylglycerol (DAG)–protein kinase C (PKC) and Janus kinase–signal transducer and activator of transcription (JAK–STAT) [11]. Activated FGFRs localize to the cytoplasm, where they undergo lysosomal degradation or the recycling process, which are partially controlled by CbL-mediated monoubiquitylation or the LIS1/NDE1 complex [25,32]. FGFR signaling may be negatively regulated by MAPK phosphatase 3, Sprouty proteins, and SeF (similar expression to FGF, also known as IL17RD) family members [33,34,35] (Figure 1). As the down-regulation of the activated receptors is an important process in order to prevent altered signaling, a defective FGFR ubiquitination system and/or an error in the mitigation pathway could induce aberrant cell growth and malignant transformation [36].

## 3. FGFR Genetic Alterations in Breast Cancer

*FGFRs’* genetic alterations may contribute to tumor progression and poor outcomes in breast cancer patients. In this regard, an analysis performed in a cohort of 391 tumor patients has evidenced that *FGF* or *FGFR* aberrations are frequent in breast cancer (32.1%) and co-exist with other aberrations. For instance, univariate analysis identified in 15 genes certain anomalies associated with *FGF/FGFR* alterations [37]. A large next-generation sequencing analysis of approximately 5000 tumors showed that *FGFR* aberrations are harbored in about 7.1% of cancers [38]. Among the *FGFR* alterations, gene amplification has been found in an elevated percentage of cases (66%), however point mutations (26%) and chromosomal rearrangements (8%) have also been detected [38]. On the basis of the aforementioned observations and considering that many *FGFR* aberrations may lead to a gain-of-function, it has been hypothesized that FGFR inhibitors would be useful as anticancer agents [39]. 

### 3.1. FGFRs Gene Amplification

The amplification of *FGFR1* represents the most frequent genomic alteration in breast cancer, whereas the amplification of the *FGFR2-4* genes is less common [40]. In particular, the *FGFR1* locus (8q12) has been found to be amplified in nearly 15% of hormone receptor (HR)-positive breast cancer and in approximately 5% of the aggressive triple-negative breast cancer (TNBC) [40]. The genomic analysis of The Cancer Genome Atlas (TCGA) and the Molecular Taxonomy of Breast Cancer International Consortium (METABRIC) databases has confirmed that the amplification of *FGFR1* is the highest among the FGFR family members, as it occurs in nearly 14% of breast cancer patients [9]. Furthermore, those patients with either a high expression of FGFR1 or copy number gain do exhibit reduced overall survival rates compared to the remaining cohort of patients [9]. It is worth mentioning that the amplification of *FGFR1* has been correlated with aberrant ligand-dependent and ligand-independent signaling, which may lead to resistance to endocrine treatments in breast cancer [41,42]. In particular, breast cancer patients with the *FGFR1* amplification frequently harbor activating alterations in the *PIK3CA* gene [38]. Likewise, *FGFR1* has been found to be co-amplified with the *CCND1* gene towards poor outcomes in estrogen-receptor (ER)+ breast cancer [23,43]. A recent study has also shown that genetic aberrations of *FGFR, TP53*, *FLT1*, and HER2+ may forecast the occurrence of brain metastases in breast tumor patients [44]. Among the *FGFR* aberrations, *FGFR1* abnormalities were the most frequent and significantly higher in patients with brain metastases with respect to the remaining cohort of patients [44]. In addition, patients with ER+ metastatic breast cancer displayed the amplification of *FGFR1* in circulating tumor DNA (ctDNA), hence mirroring the findings obtained in genomic studies of ER+ breast cancer tissues [45]. Together, these results indicate that the amplification of *FGFR1* may represent an oncogenic driver contributing to breast cancer progression. 

The amplification of the *FGFR2* locus (10q26) is a rare event in breast cancer, as it occurs in less than 1% of all cases [38]. However, the amplification and overexpression of FGFR2 have been detected in approximately 4% of TNBCs [46]. In *FGFR2*-amplified TNBC cells, a constitutive receptor activation highly sensitive to FGFR inhibition has been observed, suggesting that FGFR2 might be a potential therapeutic target in this occurrence [46,47,48]. In addition, high levels of FGFR2 have been found to be correlated with low overall and disease-free survival rates [49]. However, a further study performed on 148 TNBC samples reported that the *FGFR2* amplification did not affect the survival of patients [50]. In this context, it should be mentioned that *FGFR2* amplification may occur in normal breast tissue [20,51,52,53], making therefore uncertain the consideration of FGFR2 as a potential therapeutic target. Regarding the *FGFR3* locus (4p16), its amplification has been detected in less than 1% of patients with breast cancer. In particular, analyses performed using the TCGA and METABRIC datasets have displayed the amplification of *FGFR3* in five (0.5%) and nine (0.5%) cases, respectively [38,54]. A small frequency (2.3%) has been observed for the amplification of *FGFR4* (5q35 locus) [38]. Yet, increased FGFR4 mRNA levels have been found in up to 30% of breast cancer patients; thus, new investigations are warranted in order to better assess such discrepancy occurring between the amplification of *FGFR*4 and its mRNA levels [55]. In this context, it should be noted that a retrospective study has assessed high FGFR4 levels independently associated with the therapeutic response and the survival of ER+ breast cancer patients treated with tamoxifen [56].

### 3.2. FGFRs Point Mutations

FGFR somatic mutations, which represent 26% of FGFR aberrations, are frequently observed within the receptor ligand binding and transmembrane domains [57]. Although rare in breast cancer, certain FGFR activating mutations are able to trigger an aberrant signaling in a ligand-independent manner towards an oncogenic activity exerted in this tumor [38]. The analysis of the Catalog Of Somatic Mutations In Cancer (COSMIC) database has identified a limited number of mutations of FGFRs in breast cancer samples. In particular, FGFR1 mutations have been assessed in 2 (S125L and K566R) out of 1031 samples, an FGFR2 mutation (R203C) has been shown in 1 out of 637 samples, and an FGFR4 mutation (V550E) has been identified in 1 out of 550 samples [7]. Certain FGFRs mutations with unknown functional significance have also been identified in patients with breast cancer [58]. In addition to the abovementioned data, several studies have hypothesized that germline Single Nucleotide Polymorphisms (SNPs) in the FGFR loci may play a role in breast cancerogenesis. In this vein, the Genome-Wide-Association-Studies (GWAS) has ascertained that some SNPs (rs2981582, rs1219648, rs2420946, rs2981579) within the FGFR2 intron 2 are associated with an increased breast cancer risk in postmenopausal women [59,60,61]. An SNP in the FGFR4 transmembrane region (rs351855), which involves the substitution of a glycine (G) into an arginine (R), has been associated with a poor prognosis in solid tumors, including breast cancer [62]. Accordingly, two recent studies performed in 1492 and 747 patients have suggested that the presence of the FGFR4-R388 allele confers an increase in breast cancer risk and correlates with lymph node metastasis and poor survival [63]. This FGFR4-associated SNP is important for breast cancer susceptibility, as it causes a receptor conformational change that allows functional interaction with the signal transducer and activator of transcription 3 (STAT3) [64]. Conversely, an FGFR1-associated SNP (rs17182023) has been linked to a low FGFR1 protein expression and a reduced breast cancer risk [65]. Certain genetic variants, such as FGFR4 rs1966265 and FGFR2 rs2981578, have also been involved in the outcome of breast cancer patients treated with chemotherapeutic agents [66]. Overall, the aforementioned findings are not strong enough to suggest that these SNPs may be considered as a therapeutic target in breast cancer patients.

### 3.3. FGFRs Gene Fusions

It is well known that fusion genes derived from the rearrangement of independent genes may trigger cancer development [67]. In this regard, it has been reported that several *FGFR* translocations may generate fusion proteins by combining the N-terminus part of a transcription factor and the TK domain of FGFR1-3. These chimeric proteins dimerize constitutively, allowing the activation of the TK domain [68,69]. For instance, a chromosomal rearrangement between the endoplasmic reticulum lipid raft-associated 2 (*ERLIN2*) and *FGFR1* generates a chimeric protein, which displays a constitutive activation of the FGFR1 kinase domain, similar to that observed with the fusion of the AF4/FMR2 Family Member 3 (AFF3) with *FGFR2* [68]. In breast tumor specimens and in SUM185PE TNBC cells, an oncogenic rearrangement similar to that reported in glioblastoma and bladder cancer has been found [54]. This chimeric gene is generated by the fusion of the coiled-coil domain of transforming acidic coiled-coil 3 (*TACC3*) with *FGFR3*. The presence of the coiled-coil domain of TACC3 enhances the dimerization of the fusion protein and activates FGFR3 tyrosine kinase, which in turn stimulates tumor formation [54]. Further partners such as TACC1, TACC2, BAIAP2L1, BICC1, NPM1, PPAPDC1A, SLC45A3, and AHCYL1 should also be mentioned among the FGFR1-3 fusion genes leading to relevant biological actions [38,68].

## 4. Deregulation of FGF/FGFR Signaling across Breast Cancer Subtypes 

Breast tumors can be classified according to their molecular, histological, and clinical features [70,71,72,73]. The widely-accepted subtyping of breast cancer based on the Prediction Analysis of Microarray of 50 gene expression profiles (PAM50) includes luminal A, luminal B, HER2-enriched, basal-like, and normal breast-like [72]. The clinical subtyping of breast cancer is instead based on the immunohistochemistry detection of ER, progesterone receptor (PR), and HER2. In addition, the Ki-67 index has been included as a marker, thus classifying tumors as luminal A (ER+/HER2−/Ki67−), luminal B1 (ER+/HER2−/Ki67+), luminal B2 (ER+/HER2+), HER2+ (ER−/PR−/HER2+) and triple-negative (ER−/PR−/HER2−) [73]. In this scenario, diverse studies have demonstrated that an aberrant activation of the FGF/FGFR axis may be involved in the progression of the different subtypes of breast cancer [28,74,75,76]. 

### 4.1. Luminal A/B Breast Cancer Subtypes

Over the past two decades, several reports have suggested the existence of a functional crosstalk between FGFRs and the HR signaling pathway towards cancer progression and the failure of endocrine therapy [77]. For instance, FGF overexpression in ER+ breast cancer cells has been shown to confer estrogen-independent cell proliferation and metastatic spread [78]. FGFR1 amplification and overexpression have been frequently observed in ER+ breast tumors exhibiting an increased proliferation rate and reduced distant metastasis-free survival [42]. In this regard, it has been demonstrated that ER+ breast cancer cell lines harboring the amplification of FGFR1 rely on active FGFR1 signaling for anchorage-independent cell growth and resistance to endocrine therapy [42]. In addition, estrogen deprivation led to an increased expression of FGFR1 and FGF ligands in ER+/FGFR1-amplified primary tumors and breast cancer cells. In estrogen-free conditions, FGFR1 was associated with ER in tumor cell nuclei and regulated the transcription of ER-dependent genes. Likewise, the growth of ER+/FGFR1-amplified cells and PDXs was potently inhibited by the combination of drugs targeting both receptors [79]. Next, an adverse prognostic impact of FGFR1 expression has been assessed in the luminal A subtype of breast cancer [80]. In line with these results, FGFR1 amplification and overexpression have been shown to contribute to resistance to the CDK4/6 inhibitors used in combination with endocrine therapy in either in vitro or in vivo patient-derived xenograft models [23]. Recently, it was demonstrated that, in ER+ breast cancer cells, the activation of the FGFR1/3-STAT3 signaling pathway leads to resistance to the PI3K/mTOR inhibitor named NVP-BEZ235 [81]. Tomlinson and co-workers have also shown that the expression of FGFR3 is higher in a subset of tamoxifen-resistant ER+ breast cancers with respect to tamoxifen-sensitive ER+ breast tumors [82]. Likewise, the stimulation of FGFR3 has been found to trigger resistance to tamoxifen via the activation of the PLCγ signaling cascade [82]. It has been therefore suggested that FGFR3 may be considered as a promising target in the treatment of breast cancer patients exhibiting resistance to endocrine therapy [82]. It should be mentioned that, in breast cancer cells, the activation of the FGF7/FGFR2 axis may induce the proteasomal degradation of ER, preventing the inhibitory effects elicited by tamoxifen [83]. It is noteworthy that, in the analysis of tissue samples of patients with invasive ductal breast carcinoma, an inverse correlation between the expression of FGFR2 and ER was assessed, further supporting the aforementioned in vitro data [83]. Altogether, these findings suggest a potential association between FGFR overexpression and/or activation and acquired resistance to endocrine therapy in hormone-responsive breast cancers.

### 4.2. HER2-Enriched Subtype

An integrated molecular analysis of breast carcinoma has evidenced that HER2+ breast tumors harbor high expression levels of many receptor tyrosine kinases, including FGFR4 [84]. Experimental studies using lapatinib-resistant HER2+ breast cancer cells have found the amplification and overexpression of the FGFR2 levels [75]. Furthermore, mouse mammary tumor models have revealed that combination therapies targeting FGF and ErbB receptors, including HER2 (ErbB2), may lead to synergistic anticancer effects [76]. In this context, amplified FGFR1 signaling has been included among the mechanisms involved in acquired resistance to lapatinib+trastuzumab co-targeted therapies in HER2-enriched breast cancers [6]. Overall, these results suggest the role prompted by aberrant FGFR signaling in HER2-driven tumor development, as well as the effectiveness of combination treatments targeting both pathways.

### 4.3. TNBC Subtype

Numerous studies have focused on the identification and characterization of the aberrant signaling pathways involved in the aggressive features of TNBCs [85,86]. In this vein, the amplification of the *FGFR1* and *FGFR2* gene loci has been described as recurrent in this breast cancer subtype [38,42]. For instance, a bioinformatic analysis based on publicly available datasets has unveiled that a subgroup of TNBC patients harbors the amplification of *FGFR2* [46]. The same study has also shown that TNBC cells exhibiting FGFR2 overexpression display a high susceptibility to the apoptotic cell death prompted by the selective FGFR inhibitor PD173074 [46]. In accordance with these data, PD173074 induced either cell cycle arrest or apoptosis in a panel of breast cancer cell lines, including TNBC cells [74]. In addition, an analysis of the TCGA dataset performed on TNBC samples highlighted the association between the mRNA expression of FGFR1, FGFR2, and FGF2 and the low methylation status of the respective promoter sequences [50].

## 5. The Functional Interplay between FGF/FGFR Signaling and Breast Tumor Stroma 

Breast cancer evolution is driven not only by tumor-cell intrinsic factors but rather relies on an intricate connection between cancer cells and the surrounding stroma. In this scenario, it should be noted that the tumor stroma accounts for various cell types, such as fibroblasts, endothelial and immune cells, and adipocytes embedded in a highly dynamic glycoprotein-rich extracellular matrix [87]. Together, this complex system represents a major hurdle to better understand the multiple mechanisms hidden behind the therapeutic failure [88,89]. Therefore, to better appreciate the role elicited by FGF/FGFR signaling in breast cancer progression, unraveling its contribution to the functional interplay occurring among the key players within the tumor microenvironment may represent a primary challenge. 

### 5.1. Cancer-Associated Fibroblasts

Cancer-associated fibroblasts (CAFs), which are abundantly present in the tumor stroma, may facilitate cancer cell proliferation, migration, invasion, and angiogenesis [90,91,92]. In this respect, it should be pointed out that CAFs and their secretome (i.e., chemokines, cytokines, and growth factors) exert an important role in the acquisition of certain malignant features [93]. Of note, several studies have recognized that CAFs are a source of FGF ligands, which may regulate multiple processes acting in an autocrine and/or paracrine manner [90,94]. In this regard, the CAFs derived from hormone-independent breast cancers displayed higher levels of FGF2 than CAFs derived from hormone-dependent breast tumors [95]. It is noteworthy that the FGF2 secreted by CAFs triggered PR-mediated responses in tumor cells towards the stimulation of cell proliferation [95]. Next, CAFs may alter the beneficial effects of endocrine therapy through the involvement of the FGF/FGFR system. For instance, the overexpression of FGF4 has been shown to promote the transition from estrogen-dependent to estrogen-independent tumor growth and to trigger a metastatic phenotype in xenograft mouse models [96]. Besides this, the release of FGF7 by CAFs and its interaction with the cognate receptor FGFR2 have been shown to induce ER phosphorylation, ubiquitination, and subsequent proteasomal degradation, therefore counteracting the endocrine treatment in breast cancer cells [83]. Besides this, the FGF/FGFR axis has been involved in a feedforward stimulatory loop coupling CAFs to breast cancer cells [97,98], whereas the PDGF released by breast tumor cells stimulated the production of FGFs by CAFs towards the activation of proliferative and pro-metastatic pathways in breast cancer cells [97]. Likewise, the Hedgehog ligand produced by TNBC cells prompted the release of FGF5 by CAFs, leading to the acquisition of a chemo-resistant and cancer stem cell phenotype [99]. Recently, estrogens have been shown to mediate FGF2/FGFR1 paracrine activation through the G protein estrogen receptor (GPER), which coupled CAFs to breast cancer cells towards migratory and invasive tumor cell responses [9]. Together, these findings may extend previous studies showing that diverse FGF ligands are also secreted by normal fibroblasts [100]. 

### 5.2. Endothelial Cells

Angiogenesis is an essential process in tumor development and metastatic spread, as it constantly supplies tumor mass with oxygen and nutrients [101]. In this context, several reports have provided evidence that the endothelial cells of the surrounding tumor stroma express FGFRs that mediate the stimulatory action of FGFs [102,103]. Indeed, FGF/FGFR signaling has been demonstrated to act as an angiogenic driver, given that certain FGFs may be able to stimulate the formation of new blood vessels [28]. Additionally, the abnormal activation of the FGF/FGFR system has been reported to promote resistance to anti-VEGF therapy [104]. In this regard, preclinical data have shown an increased FGF2 expression in breast tumors treated with antiangiogenic agents, whereas a suitable antitumor activity was obtained, inhibiting both the VEGFR and FGFR-mediated transduction pathways [105]. 

### 5.3. Tumor-Infiltrating Immune Cells

Macrophages, neutrophils, myeloid-derived suppressor cells (MDSCs), and T and B lymphocytes mainly contribute to the immune tumor infiltrate, which plays an integral role in the evolution of breast cancer [106]. For instance, MDSCs that are highly up-regulated in pathological conditions such as the chronic infections and cancer may exhibit a strong immunosuppressive activity [107]. In this context, the infiltration of MDSCs during mammary tumorigenesis has been found to be decreased upon the FGFR inhibitor BGJ398 in a transgenic mouse model [108]. Likewise, the FGFR inhibition by AZD4547 reduced breast tumor-infiltrated MDSCs as well as their levels in the peripheral blood and the spleen [109]. The use of AZD4547 also prompted the recruitment of CD4(+) and CD8(+) T-cells in the tumor stroma and the spleen [109]. Noteworthy, FGFR inhibitors induced the immune modulations at least in part targeting the cytokines produced by CAFs, though the mechanisms involved remain to be fully understood [20]. To date, the action of the FGF/FGFR axis has been investigated in tumor-associated macrophages due to their peculiar action exerted in breast cancer [110]. For instance, it has been shown that the functional interplay linking the activation of FGFR1 in epithelial cells to the chemokine expression in macrophages (i.e., CXCL1 and CXCL5) may lead to mammary tumor formation and progression [111]. Moreover, FGFR1 activation in breast tumor cells promoted the release of inflammatory chemokine CX3CL1, which in turn stimulated the migration of macrophages during the initial stages of mammary tumor formation [112]. Next, the recruitment of macrophages and the angiogenic process mediated by FGFR1 were halted by the inhibition of CX3CR1 [112]. 

### 5.4. Cancer-Associated Adipocytes

Within the breast tumor microenvironment, relevant biological responses are triggered through the release of diverse mediators by cancer-associated adipocytes. For instance, the crosstalk between adipocytes and cancer cells may prompt functional changes in both cell types, thus supporting tumor progression [113]. In the diet-induced obesity xenograft model (DIOX), the expansion of the adipose tissue stimulated cancer progression through the production of FGF1 by the adipocytes and the crosstalk between FGFR and ER in the nearby breast cancer cells [114]. Of note, the weight gain and the adipocyte diameter positively correlated with (i) the levels of FGF1, (ii) FGFR1 activation in tumor cells, and (iii) estrogen-independent tumor growth [114]. It has also been reported that obese patients harbor an increased systemic concentration of FGF2 and exhibit resistance to anti-VEGF treatments [115]. Moreover, in a breast tumor mouse model obesity was associated with an increased release of FGF2 by adipocytes, whereas either the *antidiabetic* drug metformin or the inhibition of FGFR decreased the vessel density and restored the tumor sensitivity to anti-VEGF agents [115].

Altogether, these results indicate that the FGF/FGFR signaling may play a role in the functional interplay between cancer cells and the surrounding stroma towards breast cancer development, as recapitulated in Figure 1. 

## 6. Therapeutic Targeting of the FGF/FGFR Axis in Breast Cancer

On the basis of the aforementioned findings, diverse therapeutic approaches have been recently developed in order to target the FGF/FGFR transduction pathway [116,117,118]. In addition, different clinical trials have been performed. Nevertheless, FGFR-targeted therapies have not been approved up to now for the treatment of breast cancer [119]. To date, the current pharmacological strategies involving FGFRs may be grouped into three main categories according to the agents used, as reported in Table 1: (i) small-molecule multi-targeting Tyrosine Kinase Inhibitors (TKIs) that block the tyrosine kinase activity; (ii) small-molecule TKIs that selectively target the kinase domain of FGFRs; (iii) monoclonal antibodies (mAbs) that block FGFRs as well as entrapping their ligands.

### 6.1. Multi-Targeting TKI

Non-selective TKIs include diverse compounds targeting the tyrosine kinase domains of FGFR, VEGFR, and PDGFR, as these receptors are related phylogenetically and display a high homology. Some TKIs showed promising results regarding their safety and tolerability in early clinical trials [117]. For instance, nanomolar concentrations of Dovitinib (TKI258) inhibited a panel of RTKs, including VEGFR1-3, FGFR1-3, and PDGFRβ, showing an anti-cancer activity in preclinical models of breast tumor [76,120]. Dovitinib was then evaluated in association with fulvestrant in postmenopausal patients with HER2-enriched or ER+ breast cancer. Despite the promising clinical activity, this trial was ended early due to the slow enrollment of the FGF-amplified subgroup (NCT01528345) [121]. The non-selective inhibitor named Lucitanib (E3810), which blocks the activity of VEGFR1-3 and FGFR1-2, has been tested in a phase II clinical study involving metastatic breast cancer patients with or without FGFR1 amplification. However, the results of this clinical trial are not yet available (NCT02202746). The small-molecule Lenvatinib (E7080), which targets FGFR1-4, VGFR1, PDGFR, RET, and KIT, suppressed lymph node and lung metastases in a breast cancer xenograft model [122]. Currently, a phase II clinical trial regarding the use of Lenvatinib for the treatment of early-stage ER+ breast cancer patients is ongoing (NCT03168074). Next, the potent dual inhibitor—namely, 3D185, which targets FGFR1-3 and colony stimulating factor 1 receptor (CSF1R)—showed promising anti-tumor effects in FGFR-dependent and macrophage-dominant cancer models [123]. Interestingly, this study emphasized the usefulness of targeting simultaneously tumor cells and components of the cancer microenvironment in order to achieve synergistic beneficial effects [123].

### 6.2. Selective FGFR-TKI

Selective FGFR agents have been developed to appreciate the on-target FGFR inhibition in patients harboring FGF/FGFR abnormalities. In this regard, the pan-FGFR inhibitor Infigratinib (BGJ398) was first evaluated to determine its maximum tolerated dose and the pharmacokinetic and pharmacodynamics properties (NCT01004224) [124]. Thereafter, the combination of BGJ398 with the PI3Kα inhibitor BYL719 was investigated in patients whose advanced or metastatic tumors express *PIK3CA* mutations with or without FGFR1–3 alterations (NCT01928459) [125]. However, no clear evidence of synergistic activity was observed in the cohorts of patients harboring dual *PIK3CA* and *FGFR1*–3 aberrations [125]. The FGFR1–3 inhibitor named AZD4547 was tested in a phase II clinical trial in patients with advanced tumors, including HER2-negative breast cancer, non-small cell lung cancer, and gastroesophageal carcinomas harboring FGFR1/2 amplification. In this vein, treatment with AZD4547 has shown a higher antitumor activity in FGFR2-amplified gastroesophageal cancers with respect to FGFR1-amplified breast tumors (NCT01795768) [126]. The therapeutic response to AZD4547 used in combination with anastrozole or letrozole was evaluated in a phase II clinical study in ER+ breast cancer patients, however the results are not yet known (NCT01791985). Further clinical studies are ongoing in order to determine the effectiveness of other selective FGFR inhibitors. For instance, the small molecule Debio-1347 is currently used in combination with fulvestrant in a phase II clinical study aiming to assess the response in ER+ metastatic breast cancer with *FGFR* amplification (NCT03344536). Next, a phase II clinical trial has been set in order to appreciate the efficacy of TAS-120 used alone or in association with fulvestrant in patients with advanced or metastatic breast tumor harboring *FGFR* amplification (NCT04024436). In addition, the pan-FGFR inhibitor Erdafitinib, used in combination with fulvestrant and palbociclib, is currently under evaluation in a phase I clinical study to assess the anti-tumor activity in patients with ER+/HER2-/FGFR-amplified metastatic breast cancer (NCT03238196). 

### 6.3. Ligand Trap and Antibodies

Although considerable efforts have been recently addressed to the development of drugs targeting the FGFR kinase activity, further approaches evaluating the action of monoclonal antibodies (mAbs) as therapeutic agents have been considered. For instance, mAb GP369 recognizes the FGFR2-IIIb isoform -nhibited tumor burden both in vitro and in vivo models [127]. The effectiveness of the mAbs FPA144 and MFGR1877S that block, respectively, FGFR2 and FGFR3 and the usefulness of the soluble decoy receptor named FP-1039 that sequesters FGFs and inhibits FGFR signaling have also been ascertained [128,129]. Whilst these mAbs have shown promising anti-tumor effects in advanced solid tumors, including breast cancer, their clinical potential has been only partially explored [20]. FP-1039, which has been evaluated in a phase I clinical trial including breast cancer, has shown promising results in terms of its tolerability and low toxicity with respect to FGFR-TKI (NCT00687505) [130]. 

Overall, anti-FGF/FGFR agents used alone and/or in combination with established treatments have displayed an intriguing potential to be considered in comprehensive therapeutic approaches in breast cancer. 

## 7. Conclusions and Future Perspectives

The choice of a targeted therapy for each patient is currently made by analyzing the expression and/or the genomic status of a specific molecular target. Nevertheless, the heterogeneity of breast tumor cells frequently makes this decision challenging. In this context, it should be mentioned that numerous studies performed both in vitro and in vivo have evidenced that aberrant FGF/FGFR signaling may contribute to the onset and progression of breast tumor [117]. Furthermore, the overexpression and/or abnormal activation of FGFRs have been frequently associated with an acquired resistance to well-established anti-cancer therapies [20,39]. Moreover, diverse studies have documented that the FGFR signaling cascade may contribute to estrogen-independent cell growth in the endocrine-resistant breast cancer subset [41,42,77,78,79].

Several reports have also highlighted the role of the FGF/FGFR system in the functional liaison between breast cancer cells and the surrounding tumor stroma [20,21]. Preclinical and clinical studies have indeed provided important hints on the feasibility of targeting FGFR in a novel anti-tumor strategy, at least in the subset of breast cancer patients harboring *FGFR* abnormalities [23,76,116,121,123]. On the other hand, the clinical trials described may evidence that considerable attention has been recently shifted to the usefulness of anti-FGFR drugs in combination with other chemotherapeutic agents [131]. Building up more knowledge of the FGF/FGFR pathway, these findings may represent the main goal in further studies aimed at addressing innovative anti-cancer strategies. In this vein, it would be critical to identify a sub-population of patients harboring peculiar molecular alterations associated with the dysregulation of the FGF/FGFR pathway towards novel co-targeting therapeutic strategies. Likewise, it remains crucial to better dissect the mechanisms involved in the action of FGF/FGFR signaling in breast cancer development in order to achieve therapeutic advances and overcome treatment hindrances. For instance, some FGFR alterations are potential targets for monotherapy, such as FGFR1-2 amplification or FGFR3 fusion genes. Other FGFR aberrations do not seem to be suitable biomarkers, hence remaining to be carefully evaluated in breast cancers patients. 

Compelling evidence has suggested that intra- and inter-tumoral heterogeneity should be taken into careful consideration for an adequate selection of patients [2,132]. In this respect, liquid biopsy allowing the identification of circulating tumor cells (CTCs) in the peripheral blood and the sequencing of the tumor DNA extracted from CTCs would provide the genetic profile of the malignancy [133]. As several studies have unveiled comparable mutation frequencies between the genomic profile of breast cancer biopsies and ctDNA [134,135,136], the assessment of the FGFR aberrations in ctDNA might be a promising strategy to monitor the sensitivity or resistance to therapies, hence facilitating patient-tailored precision medicine [45,137,138]. 

Overall, we are only at the beginning of truthfully appreciating the role played by the FGF/FGFR transduction pathway in breast malignancy, as well as how it may be halted in a suitable manner. Thus, further studies are warranted in order to gain valuable insights into the treatments targeting the FGF/FGFR system in the diverse subpopulation of breast cancer patients.

## Figures and Tables

**Table 1:** Summary of the selected FGFR inhibitors investigated in breast cancer clinical trials.

Agent	Target/s	Clinical Trial, Identifier Code	Phase	Status
**Non-selective inhibitors**				
Dovitinib (TKI258)	FGFR1-3 VEGFR1-3, PDGFRβ	NCT01528345	II	Terminated early (Slow and low enrollment)
Lucitanib (E3810)	FGFR1-2 VEGFR1-3	NCT02202746	II	Completed
Lenvatinib (E7080)	FGFR1-4, VGFR1, PDGFR, RET and KIT	NCT03168074	II	Recruiting
**Selective inhibitors**				
Infigratinib (BGJ398)	FGFR1–4	NCT01004224 NCT01928459	I I	Completed Completed
AZD4547	FGFR1–3	NCT01795768 NCT01791985	II I/II	Unknown Completed
Erdafitinib (JNJ-42756493)	FGFR1–4	NCT03238196	I	Recruiting
Debio-1347	FGFR1–3	NCT03344536	I/II	Recruiting
TAS-120	FGFR1–4	NCT04024436	II	Recruiting
**Ligand trap and antibodies**				
FPA144	FGFR2	NCT02318329	I	Completed
FP-1039	FGF1, 2, 4	NCT00687505	I	Completed

## Abbreviations

FGF	Fibroblast Growth Factor
FGFR	Fibroblast Growth Factor Receptor
RTKs	Receptor tyrosine kinases
ER	Estrogen Receptor
PR	Progesterone Receptor
EMT	Epithelial to mesenchymal transition
HPSGs	Heparan sulfate proteoglycans
FRS2	Fibroblast Growth Factor Receptor substrate 2
MAPK	mitogen-activated protein kinase
PI3K	phosphoinositide-3-kinase
DAG	diacylglycerol
PIP3	phosphatidylinositol (3,4,5)-triphosphate
IP3	inositol-1,4,5-trisphosphate
PKC	protein kinase C
PLCγ	Phospholipase Cγ
CbL	Casitas B-lineage Lymphoma
LIS1/NDE1	Lissencephaly1/nudE neurodevelopment protein 1
JAK	Janus kinase
STAT	signal transducer activator of transcription
SeF	similar expression to FGF
CRISPR	Clustered Regularly Interspaced Short Palindromic Repeats
TNBC	Triple-negative breast cancer
SNPs	single nucleotide polymorphisms
GWAS	Genome-Wide-Association-Studies
ERLIN2	ER Lipid Raft Associated 2
AFF3	AF4/FMR2 Family Member 3
TACC	transforming acidic coiled-coil
BAIAP2L1	BAR/IMD Domain Containing Adaptor Protein 2 Like 1
BICC1	BicC Family RNA Binding Protein 1
NPM1	Nucleophosmin 1
PPAPDC1A	phosphatidic acid phosphatase type 2 domain containing 1A
SLC45A3	Solute carrier family 45 member 3
AHCYL1	Adenosylhomocysteinase Like 1
HER2	human epidermal growth factor receptor 2
CDK4/6	Cyclin-dependent kinase 4/6
CAFs	Cancer Associated Fibroblasts
VEGF	Vascular Endothelial Growth Factor
VEGFR	Vascular Endothelial Growth Factor receptor
PDGF	Platelet-Derived Growth Factor
PDGFR	Platelet-Derived Growth Factor receptor
GPER	G-protein estrogen receptor
MDSCs	Myeloid-derived suppressor cells
CXCL	C-X-C motif chemokine ligand
CX3CL1	C-X3-C motif chemokine ligand 1
CX3CR1	C-X3-C motif chemokine receptor 1
TKIs	Tyrosine Kinase inhibitors
mAbs	Monoclonal antibodies
HRs	Hormone receptors
CSF1R	Colony Stimulating Factor-1 receptor
FGF	Fibroblast Growth Factor
